# Surgical treatment of Shone’s syndrome and patent ductus arteriosus in an adult

**DOI:** 10.1186/s12872-022-02991-1

**Published:** 2022-12-07

**Authors:** Yanzhong He, Yang Jiang, Feng Wan, Xiaodong Feng, Yifei Hua

**Affiliations:** 1 Department of Cardiovascular Surgery, Shanghai East Hospital, Tongji University School of Medicine, Shanghai, 200120 China; 2 Research Center for Translational Medicine, Shanghai East Hospital, Tongji University School of Medicine, Shanghai, 200120 China

**Keywords:** Shone’s syndrome, Coarctation of the aorta, Parachute mitral valve, Patent ductus arteriosus

## Abstract

**Background:**

Shone’s syndrome is a rare complex congenital anomaly. The classical definition consists of four anomalies: supravalvular mitral membrane, parachute mitral valve (PMV), subaortic stenosis, and coarctation of the aorta (CoA). Few studies have been reported on Shone’s syndrome in adults, particularly the primary surgical correction of the anomalies.

**Case presentation:**

A 22-year-old female patient presented with chest distress and tachypnea. Echocardiography and CT revealed supravalvular mitral membrane, PMV, Bicuspid aortic valve stenosis, CoA and patent ductus arteriosus. She underwent primary definitive surgical correction successfully and was discharged from hospital with symptoms free.

**Conclusions:**

Our case report highlights the importance of echocardiographic evaluation in the diagnosis of Shone’s syndrome. The surgical strategy should be tailored according to both the patient’s profile and the surgeon’s personal surgical experience. Extra-anatomical bypass procedure is an appropriate technique for adult patients with long-segment coarctation and concomitant cardiac lesions. The outcomes of the case study indicate that the primary definitive surgery is encouraging.

## Background

Shone’s syndrome, first described by Shone in 1963, is a very rare complex congenital heart disease. It is characterized by four obstructive cardiac anomalies of the systemic circulation, including supravalvular mitral membrane, parachute mitral valve (PMV). subaortic stenosis and coarctation of aorta (CoA) [1]. Echocardiography plays an important role in the diagnosis and is the optimal examination for detecting this disease. Few studies have been reported on Shone’s syndrome in adults, particularly the primary surgical correction of the anomalies [2, 3].

Here, we present a case of Shone’s syndrome and patent ductus arteriosus (PDA) in a 22-year-old patient, underwent operation in the Department of Cardiovascular Surgery, Shanghai East Hospital in July, 2020.

## Case presentation

### History

A 22-year-old female patient presented with chest distress, tachypnea associated with dizziness, headache, and cold legs for 5 months.

### Physical examination

The lower limbs were cold, and pulsation of dorsalis pedis artery was absent. The blood pressure in the upper and lower extremities was 180/92 mm Hg and 110/84 mm Hg, respectively. A grade 3/6 continuous murmur was audiable near the left sternal border at the second (L2) intercostal space, and a grade 2/6 diastolic murmur at the apex.

### Echocardiography examination

**Fig. 1:**
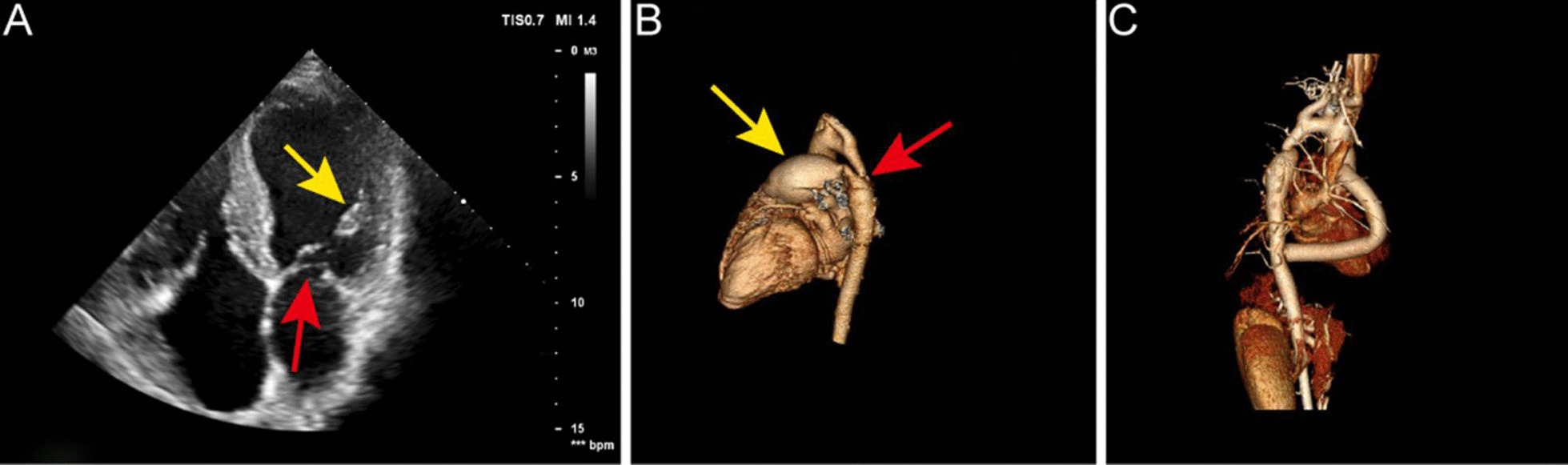
Echocardiography and CT examination reports. **A** Supravalvular mitral membrane attached to the wall of the left atrium (red arrow) and solitary papillary muscle (yellow arrow). **B** CT of patent ductus arteriosus. A localized narrowing was observed at the aortic arch near the descending aorta (red arrow) and pulmonary artery dilatation (yellow arrow). **C** Prosthetic bypass between the ascending and the descending aorta

Echocardiography showed supravalvular mitral membrane attached to the wall of the left atrium and couple chordae tendineae attached to the solitary papillary muscle (Fig. 1A). A parachute deformity of the mitral valve was detected and the effective orifice area (EOA) of the stenotic valve was 0.55 cm^2^. Bicuspid aortic valve (BAV) with right-left coronary cusp fusion and moderate aortic stenosis with a peak systolic velocity and a gradient peak pressure of 3.4 m/s and 46.2 mm Hg respectively, were observed. The aortic valve regurgitation was mild and no subaortic stenosis was detected. The diameter of aortic coarctation segment is approximately 7.0 mm, with a peak systolic velocity and a gradient peak pressure of 4.2 m/s and 70.6 mm Hg, respectively. A 9.0 mm tubular PDA was observed. The pulmonary artery systolic pressure was 106 mm Hg.

### Imaging examination

Computed tomography (CT) showed CoA and PDA (Fig. 1B). On right-heart catheterization, the pulmonary vascular resistance elevated to 9.27 Wood units, and pulmonary artery pressure to 96/37(57) mm Hg.

### Surgical procedure

**Fig. 2:**
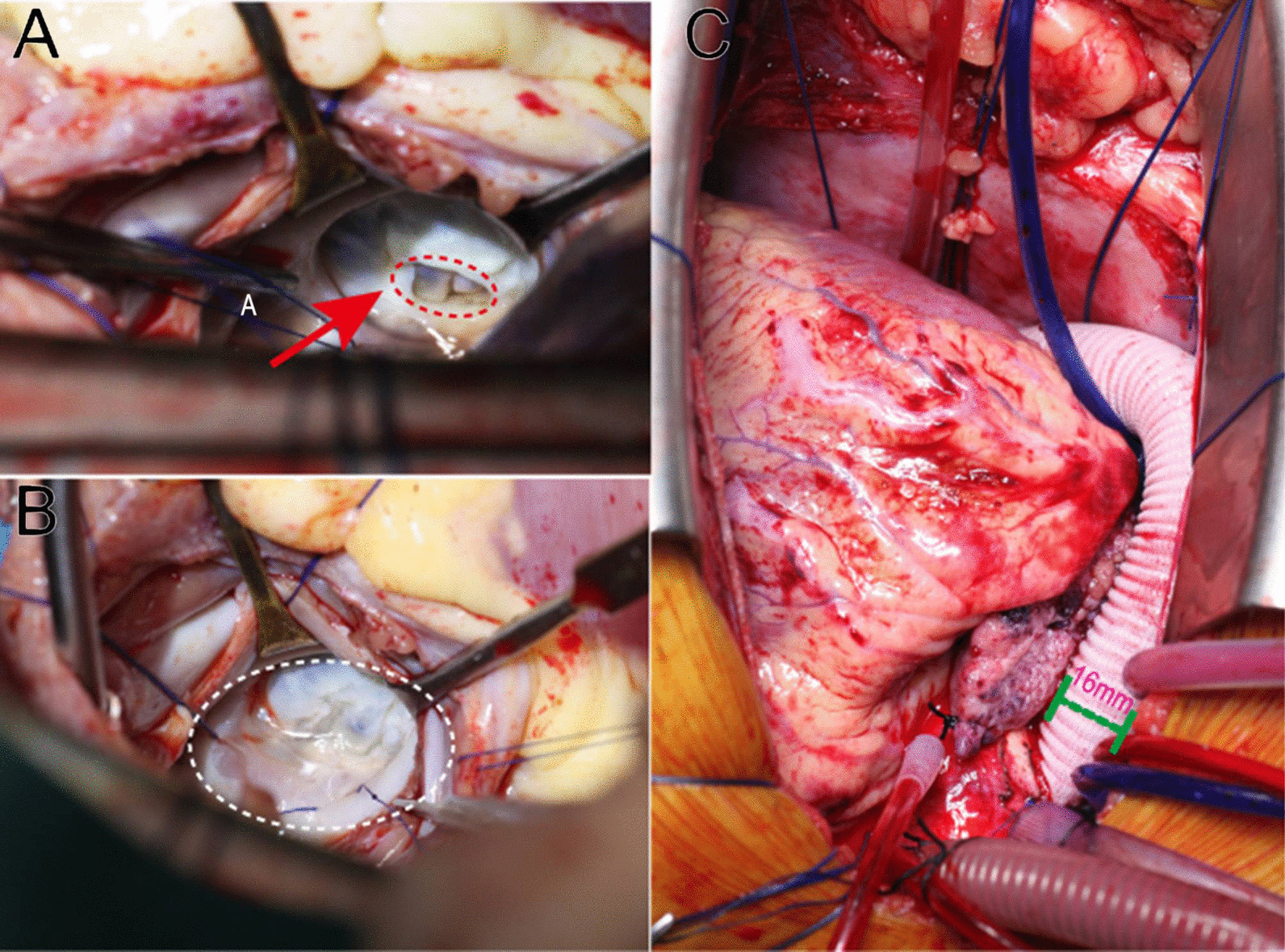
Surgical procedure. **A** Supravalvular mitral membrane (red arrow): the inner diameter of the membrane was about 0.8 cm. **B** Valve orifice area increased significantly. **C** Vascular prosthetic bypass

A median sternotomy was performed as usual. The PDA was doubly ligated with silk suture just prior to cardiopulmonary bypass. After cardioplegic arrest, the mitral valve was exposed through the right atriotomy and atrial septum (Fig. 2A). The supravalvular mitral membrane was resected and split to the posterior commissure. The valve was enlarged to admit a 19-mm Hegar dilator. The commissural fusion of left and non-coronary cusps was detected and dissected 2 mm to the aortic annulus. The descending aorta was exposed by opening the pericardium of the posterior mediastinum. A 16 × 300 mm of a straight vascular prosthesis (Type: 733016) was placed between the ascending and the descending aorta (Fig. 2B).

### Results

After the surgery, the upper-limb arterial pressure dropped to 112/63(77) mm Hg. The lower-limb arterial pressure was 87/58(70) mm Hg. Transesophageal echocardiography (TEE) demonstrated that the mitral valve orifice area increased significantly to 2.0 cm^2^ with trivial regurgitation and the aortic valve had mild stenosis and insufficiency. The skin temperature of upper and lower extremities was similar. Pulsation of the dorsalis pedis artery was evident, and headache disappeared completely. The postoperative echocardiographic data before discharge confirmed complete correction. The mitral and aortic valve openings were significantly improved compared with that before. The mitral valve orifice area was calculated to approximately 2.0 cm^2^, and the gradient peak pressure was 15 mm Hg, with a mean gradient pressure of 10 mm Hg. The aortic valve insufficiency was still mild. The gradient peak pressure of aortic valve dropped to 24 mm Hg, with a mean gradient pressure of 13 mm Hg. The pulmonary arterial systolic pressure was 50 mm Hg. No residual shunt was observed between the descending aorta and the pulmonary artery. 3D CT showed prosthetic bypass between the ascending and the descending aorta (Fig. 1C).

## Discussion and conclusions

Shone’s syndrome is a complex left-sided cardiac anomaly which consists of supravalvular mitral membrane, PMV, subaortic stenosis, and CoA. In the literature, most reports were among the childhood population, while few cases of adult patients had been described. However, the partial Shone’s syndrome, characterized by two or three of the obstructive components had been reported more rarely in adults [4, 5]. In our case, the patient had supravalvular mitral membrane, PMV, CoA and BAV stenosis, without subaortic stenosis. BAV disease is known to coexist with other congenital vascular malformations, the most common of which is CoA. Prior autopsy examination showed the incidence of CoA patients with BAV was 46% [6, 7]. There are a number of syndromes whose cardiac involvement includes BAV and left-sided obstructive lesions: Shone’s syndrome, Williams syndrome with supravalvular stenosis, and Turner syndrome with CoA [8].

Echocardiography is an effective and non-invasive method to diagnose Shone's syndrome. Patients with Shone’s syndrome usually can be diagnosed with careful echocardiographic evaluation during childhood. And as the patient ages, it becomes symptomatic. This patient was delayed in diagnosis and treatment because she lived in undeveloped west areas of China with limited access to echocardiography.

Mitral valve obstruction due to PMV may be the most critical abnormality determining the longterm outcome. The key to successful surgery is the management of the mitral valve [9]. Most of the reasons for the reoperation are mitral valve related. In this case study, the lesion of mitral valve was not very severe. Only the supravalvular mitral membrane was resected and separated from the posterior commissure. The valve admitted a 19-mm Hegar’s dilator. No obvious reflux was observed in the flushing test. TEE demonstrated a valve orifice area of 2.0 cm^2^ with trivial regurgitation. The freedom of mitral valve cusp mobility deserved no further intervention on the mitral valve.

Management of aortic coarctation in Shone’s syndrome is also vital [10]. Mutiple surgical techniques have been applied for CoA repair. Surgical repair has traditionally been the mainstay of treatment for CoA correction despite advances in endovascular technology with stents allow for minimally invasive approaches in older children and adults with native CoA and complications [11]. The resection and graft interposition technique were first described by Gross in 1951 [12, 13]. A tube graft is sewn into the aorta after the cross-clamping of the aorta and resection of the coarctate segment. It is useful for patients with long-segment CoA. However, longer cross-clamp time is required and the graft cannot grow with the patient thus being not suitable for children. A surgical follow-up study showed that all the interposition grafts dilated up by 50% of their original size for more than a decade [14]. Extra-anatomical bypass technique is an alternative strategy particularly for patients with long-segment coarctation, and concomitant cardiac procedures such as coronary artery bypass grafting or aortic valve replacement [15, 16]. It is performed through median sternotomy in adults and provides additional blood flow to the distal aorta leaving the coarctate region of aorta in situ [17]. In our case of Shone’s syndrome, the procedure is more convenient and safer for surgeons to correct all the cardiac malformations in the same incision, and in a single-stage.

The descending aorta anastomosis is deep behind the heart, and the ideal method is to expose the operative field after extracorporeal circulation. In the case study, this procedure was performed after heart rebeating to shorten the clamping time and reduce the ischemic myocardial damage. However, technically, this approach is more challenging as the partial occlusion clamp on the aorta tends to slip with the resumed heart beat and also because of the risk of fatal bleeding. The procedure needs experienced surgeons to overcome these difficulties and obstacles and achieve the goal.

In conclusion, this case study highlights the importance of echocardiographic evaluation in the diagnosis of Shone’s syndrome. The surgical strategy should be tailored according to both the patient’s profile and the surgeon’s personal surgical experience with better recognition of Shone’s syndrome. Extra-anatomical bypass procedure is an appropriate technique for adult patients with long-segment coarctation and concomitant cardiac lesions. The outcomes of the case study indicate that the primary definitive surgery of Shone’s syndrome is encouraging.

## Data Availability

All relevant data supporting the conclusions of this article are included within the article.

## Abbreviations

PMV: Parachute mitral valve
CoA: Coarctation of the aorta
PDA: Patent ductus arteriosus
EOA: Effective orifice area
BAV: Bicuspid aortic valve
CT: Computed tomography
TEE: Transesophageal echocardiography
